# A generalization of the standard map and its statistical characterization

**DOI:** 10.1038/s41598-022-12213-5

**Published:** 2022-05-20

**Authors:** Kivanc Cetin, Ugur Tirnakli, Bruce M. Boghosian

**Affiliations:** 1 Department of Physics, Faculty of Science, Ege University, Izmir, 35100 Turkey; 2 American Collegiate Institute, Izmir, 35290 Turkey; 3 Department of Mathematics, Tufts University, Medford, MA 02155 USA

**Keywords:** Statistical physics, thermodynamics and nonlinear dynamics, Statistical physics

## Abstract

From the statistical mechanical point of view, area-preserving maps have great potential and importance. These maps exhibit chaotic and regular behavior separately or together in the available phase space as the control parameter changes. Several works on these maps, e.g., the standard map and the web map, have shown that ergodicity breakdown causes the statistical mechanical framework that describes the dynamics of the system to change. In this paper, for a novel generalization of the standard map, which we define by generalizing the periodic function used in its definition, we verify that a *q*-Gaussian with $q\simeq 1.935$ for the probability distribution of sum of the iterates of the system with initial conditions chosen from the nonergodic stability islands is robust. We also show that the probability distributions become more complicated and unexpected limiting behavior occurs for some parameter regimes.

## Introduction

It is well-known that the Boltzmann-Gibbs (BG) statistical mechanical framework is able to describe the dynamics of systems that exhibit ergodicity and mixing. For the strongly chaotic regime that presents the largest positive Lyapunov exponent (LLE), the system is ergodic and the probability distribution of the sum of the appropriate variables of the system appears to be a Gaussian, indicating the validity of BG statistics^1^. On contrary, BG statistics is problematic for the description of the dynamics of nonergodic systems. In recent years it has been shown that for several dissipative^2–4^ and conservative^5–7^ model systems nonextensive statistical mechanics is a good candidate to describe systems in the weakly chaotic regime with nearly zero LLE. In that regime, probability distributions seem to be *q*-Gaussians, indicating the validity of the nonextensive statistical mechanical framework. In addition to the weakly chaotic regimes of these model systems, *q*-Gaussians have also been observed in several experimental systems such as the Couette-Taylor flow in a fully developed turbulent regime^8,9^, LHC experiments^10^, the ozone layer^11^ and dissipative optical lattices^12,13^, among many other examples in the literature that are discussed in^14^.

In recent papers, a full statistical mechanical characterization of the paradigmatic area-preserving standard map^6^ and the web map^7^ has been discussed by means of *q*-triplets^15^. It is seen that the results obtained in these studies are consistent with the results of Ref.^15^, and a stationary distribution index $q_{stat}$ obtained for the stability islands of these systems is exactly the same as the one obtained in^5^. In the limit distribution analyses, it has been observed that for large control parameter values, where the entire phase space of the map is dominated by chaotic trajectories, the probability distribution of the sum of iterates of the map is Gaussian. This observation is consistent with the BG statistical mechanical framework. On the other hand, when the phase space is entirely dominated by the stability islands (nearly zero LLE), as it is for small parameter values, the probability distribution appears to approach a *q*-Gaussian with $q\simeq 1.935$. The numerical value proposed in recent works^5–7^ agrees with the theoretical prediction made in Ref.^16^, within only one significant digit. A suitable description of the dynamics of the map for this case may therefore be provided by nonextensive statistical mechanics. For parameter values between these two extreme cases, where the chaotic regions and the stability islands coexist in the phase space, it has been shown that the probability distribution throughout the phase space is well fitted by a linear combination of a Gaussian and a *q*-Gaussian with $q\simeq 1.935$. As these observations are valuable from statistical mechanical viewpoint, they also have physical importance. Both area-preserving maps are equivalent to an iterative form of the Hamiltonian function for the kicked oscillator model, i.e., the *Q*-folded web map can be reduced to the standard map (kicked rotor) in specific limits^17^. In addition to their role in the development of the theory of nonintegrable Hamiltonian systems^18^, many physical systems of practical importance are frequently considered as a combination of these maps^19,20^. The standard map in particular has been widely used in diverse fields of physics, e.g., particle dynamics in accelerators^21^, comet dynamics^22^, autoionization of molecular Rydberg states^23^, and so on.

Inspired by its extensive usage in physics and its theoretical importance in chaos theory, we define here a new generalization of the standard map which also generalizes the Hamiltonian of the kicked rotor system. This generalization enables us to define unique maps with different phase space dynamics. As the standard map can be considered as a simple model for many physical systems, this newly defined generalization is thought to be explanatory and appropriate for more complex systems that cannot be reduced to the original standard map as a first approximation. In this paper, for several scenarios of the generalized standard map, we investigate the phase space behavior and limit distribution of initial conditions chosen from the entire phase space. With these investigations, we aim to present the robustness of the results obtained for the limiting behavior of initial conditions chosen from the stability islands of area-preserving maps.

## Methods

### Generalization of the standard map

**Figure 1:**
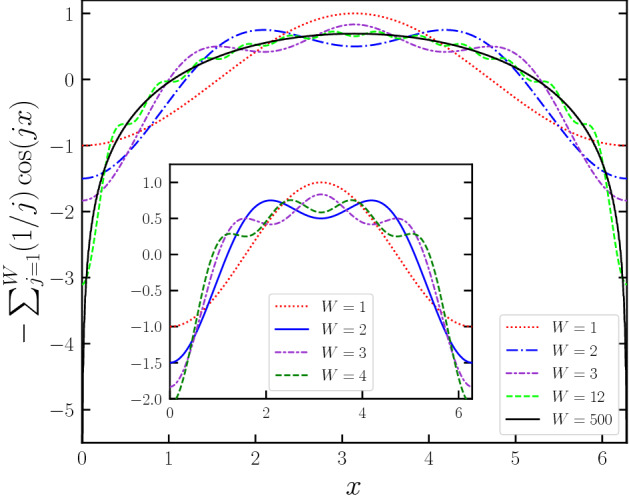
The behavior of the new potential of the generalized standard map discussed here for several representative values of *W*. The inset of the graph presents the conversion of minima and maxima with increasing *W*. In each increment step, newly arisen maxima can be clearly seen.

In similar fashion to another generalization of the standard map, namely the *z*-generalized standard map^24^, we define a new generalization of the standard map^25^ by modifying the trigonometric term in the equations that describe the system. In contrast to the *z*-generalized version, our modification is made by replacing the sine function by a summation of sine functions. This basically amounts to changing the potential term in the Hamiltonian of the kicked rotor and rewriting this Hamiltonian as

1 $$\begin{aligned} H = \frac{p^2}{2} - K \left[ \sum _{j}^{W} \frac{1}{j} \cos (jx)\right] \sum _{i}\delta (t-i\tau ) \end{aligned}$$

where *K* is a positive dimensionless map parameter that controls nonintegrability of the system, *W* is an integer, and periodic Dirac $\delta$-function models periodic kicks. The general tendency of the behavior of this potential is depicted in Fig. 1 for representative values of *W*. It is evident that, as *W* increases, the number of minima and maxima also increases, which is directly related to the value of *W*. Therefore, the proposed generalization can be considered as a *W*-well potential. The intriguing new property seen in this generalization is that, as *W* is increased, interconversion between minima and maxima is observed. With each increment in *W*, in addition to the occurrence of new maxima closer to $x=0$ and $x=2\pi$, minima of the previous potential well turn into maxima in the new well and vice versa. Moreover, as $W\rightarrow \infty$, the distance between the maxima and minima decreases. Since there will be a point for which the numerical resolution cannot locate maxima and minima correctly, the kicking potential will then become a harmonic potential. Multiple-well potentials are indeed an interesting area also available in the literature (see for example Refs.^26–33^).

Using this Hamiltonian, the generalized standard map can be given as

2 $$\begin{aligned} \begin{array}{l} p_{i+1} =p_{i}-K \sum _{j=1}^{W} \sin (jx_{i}) \\ x_{i+1}=x_{i}+p_{i+1} \end{array} \end{aligned}$$

where *x* and *p* are taken as modulo $2\pi$. At this point, it is worth mentioning that the proposed generalized force can also be written in a closed form as

3 $$\begin{aligned} -K\sum _{j=1}^{W} \sin (jx_i) = -K\sin \left[ \frac{(W+1)x_i}{2} \right] \sin \left( \frac{Wx_i}{2}\right) \csc \left( \frac{x_i}{2}\right) \end{aligned}$$

For different values of the generalization term *W*, we actually create unique area-preserving systems, each having different dynamical properties. It is easy to verify that the area-preserving nature of the map holds for this generalization. For a constant *K* value, due to the conversions and occurrences of stable and unstable points in the phase space, as *W* varies, the phase space dynamics of the trajectories change significantly.

**Figure 2:**
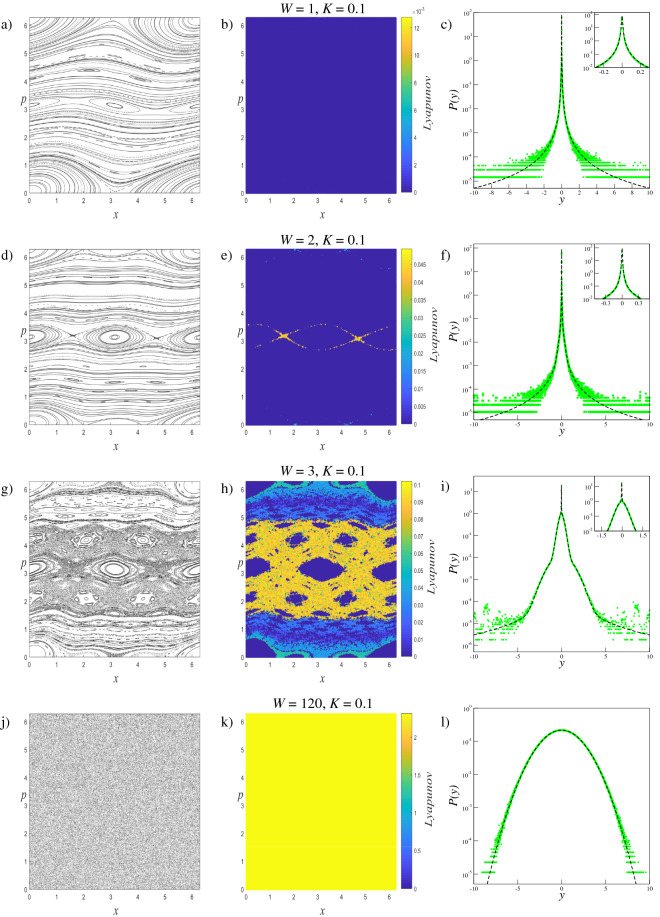
Analyses of the $W=1$, $W=2$, $W=3$, and $W=120$ systems for $K=0.1$. Left column: (**a**,**d**,**g**,**j**) Phase space portraits, Middle column: (**b**,**e**,**h**,**k**) LLE color map descriptions of the phase portraits, Right column: (**c**,**f**,**i**,**l**) Probability distributions obtained from the initial conditions chosen from the entire phase space.

**Figure 3:**
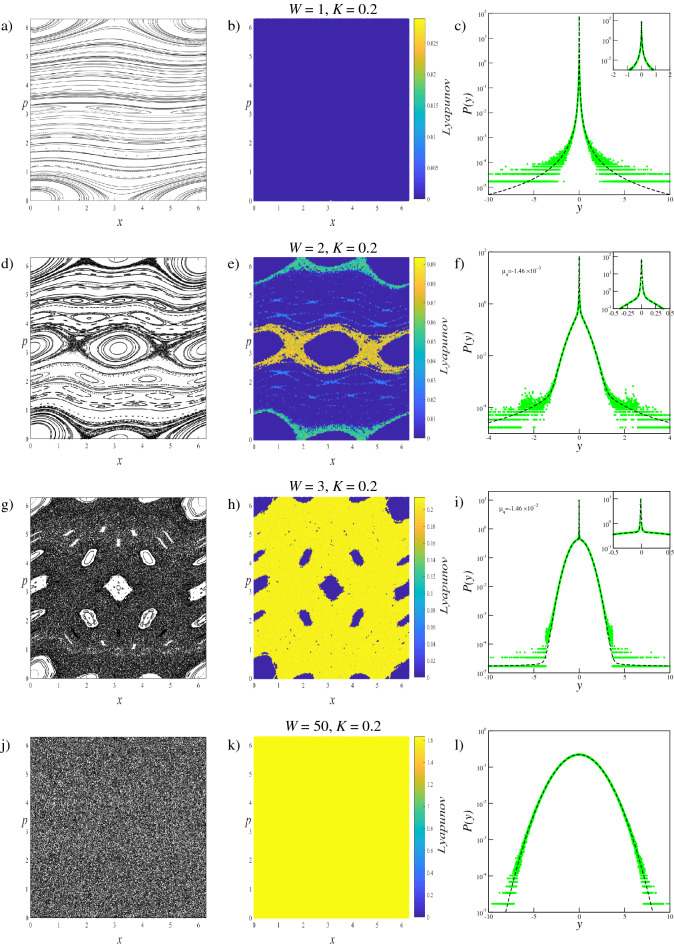
Analyses of the $W=1$, $W=2$, $W=3$, and $W=50$ systems for $K=0.2$. Left column: (**a**,**d**,**g**,**j**) Phase space portraits, Middle column: (**b**,**e**,**h**,**k**) LLE color map descriptions of the phase portraits, Right column: (**c**,**f**,**i**,**l**) Probability distributions obtained from the initial conditions chosen from the entire phase space.

**Figure 4:**
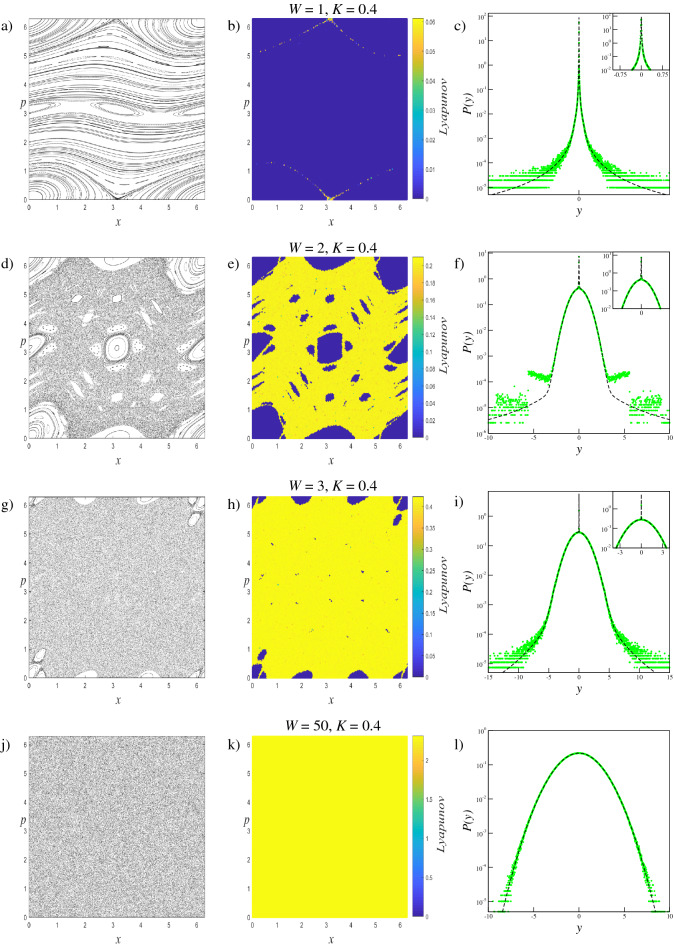
Analyses of the $W=1$, $W=2$, $W=3$, and $W=50$ systems for $K=0.4$. Left column: (**a**,**d**,**g**,**j**) Phase space portraits, Middle column: (**b**,**e**,**h**,**k**) LLE color map descriptions of the phase portraits, Right column: (**c**,**f**,**i**,**l**) Probability distributions obtained from the initial conditions chosen from the entire phase space.

**Figure 5:**
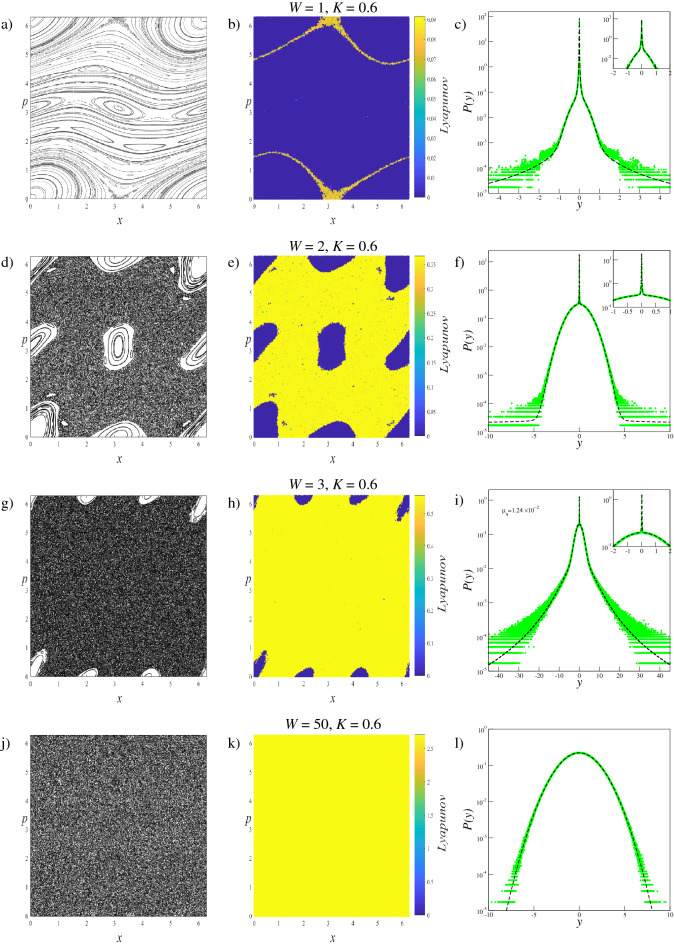
Analyses of the $W=1$, $W=2$, $W=3$, and $W=50$ systems for $K=0.6$. Left column: (**a**,**d**,**g**,**j**) Phase space portraits, Middle column: (**b**,**e**,**h**,**k**) LLE color map descriptions of the phase portraits, Right column: (**c**,**f**,**i**,**l**) Probability distributions obtained from the initial conditions chosen from the entire phase space.

We investigate the phase space dynamics of the trajectories and limiting distributions of generalized systems with $W=1$, $W=2$, $W=3$, $W=50$ and $W=120$, for $K=0.1$, $K=0.2$, $K=0.4$ and $K=0.6$ parameter values. The $W=1$ case corresponds to the original standard map, and the $W=50$ and $W=120$ parameters are selected to provide extreme scenarios in which the phase spaces are fully occupied by the chaotic sea. With this parameter set, the changes in the phase space dynamics and limiting distributions can be compared and analyzed. Results obtained fromthese analyses are given collectively for each parameter value to provide better visualization of the evolution of the system. To display how the trajectory behavior changes in the phase space according to the increment of the *W* term, for each scenario we iterate the system $T=5 \times 10^{3}$ times starting from $M=150$ initial conditions randomly chosen over the whole phase space. The small number of initial conditions are chosen for better visualizations of the phase space portraits which are provided in the first columns of Fig. 2 for $K=0.1$, Fig. 3 for $K=0.2$, Fig. 4 for $K=0.4$, and Fig. 5 for $K=0.6$.

### Lyapunov exponent analysis

To quantify the trajectory behavior in the phase space, the Benettin algorithm^34^ is used to calculate the LLE ($\lambda$) which is defined as

4 $$\begin{aligned} \lambda =\frac{1}{T}\sum _{i=1}^{T}\ln \left( \frac{d(i)}{\sqrt{2}\, \Delta _{x}^{(0)}}\right) , \end{aligned}$$

where *T* is the iteration time and $d(i)=\left[ (\Delta _{x}^{(i)})^{2}+(\Delta _{p}^{(i)})^{2}\right] ^{1/2}$ is the Euclidean distance at time *i* in the phase space between initially neighboring trajectories. For the scenarios mentioned above, the LLE is calculated by using $T=5\times 10^{5}$ iteration steps for each of $M=2.5\times 10^{6}$ initial conditions, that are randomly chosen from the entire phase space, separately. Here the initial distance between a randomly chosen initial condition and its neighbor is set as $\Delta _{x}^{(0)}=\Delta _{p}^{(0)}=10^{-8}$. Computed magnitudes of the LLE are illustrated by a color map to quantify the trajectory behavior in the phase space portrait correspondingly. This method is similar but not exactly the same as the method used so far in the literature known as finite time Lyapunov exponent^35–38^. As can be seen from the second columns of Figs. 2, 3, 4, and 5, the calculated LLE magnitudes are nearly zero ($\lambda \approx 0$) for initial conditions located inside the stability islands, whereas they are largely positive for the chaotic trajectories. Compatibly with their LLE magnitudes, the stability islands are said to be in a weakly chaotic regime^5^. With this visualization, regions of different behavior can be distinguished in the phase space. It is important to note that in LLE color maps, some tiny stability islands placed in the chaotic sea are difficult to distinguish since they are shrouded by chaotic initial conditions. This problem arises only from the point size used for initial conditions in plots and has nothing to do with the calculation process.

As seen in Figs. 2, 3, 4, and 5, for a constant *K* value, chaotic behavior dominates larger phase space areas with increasing *W* values. The underlying reason for this behavior is that the increment of the generalization term brings about the dissolving of the stability islands, which are constant energy tori, into elliptic and hyperbolic points series depending on their winding numbers forward for that *K* value. The dissolution of the tori into the elliptic-hyperbolic points series is described by the Poincaré-Birkhoff Theorem^39^. With this inference, one can say that increasing *W* increases the nonintegrability of the system for a fixed *K* value. Due to the added sine terms in the map function, resonances causing tori to dissolve according to the KAM Theorem^40^ become stronger and chaotic behavior arises from the homoclinic and heteroclinic tangles that develop by complex organization of the in-sets and out-sets of the hyperbolic points^41^. Therefore chaotic behavior can occur in the phase space at smaller *K* values for $W>1$ systems than for the original standard map. Phase space locations of the elliptic and hyperbolic points change as *W* is changed and this fact makes each generalized system and its phase space dynamics unique^42^. For the extreme cases of $W=50$, and $W=120$, the entire phase space is occupied by a single chaotic sea as a result of the large amount of a nonintegrability of the system.

### Statistical analysis

As the phase space dynamics provides rich observations for different *W* values, we can analyze the limiting distributions of these systems. Within the framework of the Central Limit Theorem (CLT) and in conformance with previous analyses of the standard map^5,6,24^ we define the variable

5 $$\begin{aligned} y = \frac{1}{ \sqrt{T} }\sum _{i=1}^{T}(x_{i}- \langle x \rangle ) \end{aligned}$$

where *T* is number of iterations. In Eq. (5), $\langle \dots \rangle$ denotes both time average over *T* iterations and ensemble average over *M* chosen initial conditions, i.e.,

6 $$\begin{aligned} \langle x\rangle = \frac{1}{M}\frac{1}{T}\sum _{l=1}^{M}\sum _{i=1}^{T}x_{i}^{(l)} . \end{aligned}$$

By defining the variable *y* in accordance with the CLT, the probability distributions are obtained as independent of the number of iterations used in the numerical calculations. It was recently shown for weakly chaotic regimes of several dissipative^2,3^ and area-preserving maps^5–7^ that limit distributions of the sum of iterates of the map (Eq. (5)) seem to approach a *q*-Gaussian form which can be defined as

7 $$\begin{aligned} P_{q}(y;\mu _{q},\sigma _{q})=A_{q}\sqrt{B_{q}}[1-(1-q)B_{q} (y-\mu _{q})^{2}]^{\frac{1}{(1-q)}} \end{aligned}$$

where $A_{q}$ is a normalization factor, $B_{q}$ is the parameter which characterizes the width of the distribution, $\mu _{q}$ is the *q*-mean value and $\sigma _{q}$ is the *q*-variance^43^:

8 $$\begin{aligned}&A_q=\left\{ \begin{array}{lc}\displaystyle \frac{\Gamma \left[ \frac{5-3q}{2(1-q)}\right] }{\Gamma \left[ \frac{2-q}{1-q}\right] }\sqrt{\frac{1-q}{\pi }}, &{}q<1\\ \displaystyle \frac{1}{\sqrt{ \pi }},&{}q=1\\ \displaystyle \frac{\Gamma \left[ \frac{1}{q-1}\right] }{\Gamma \left[ \frac{3-q}{2(q-1)}\right] } \sqrt{\frac{q-1}{\pi }}, &{}1<q<3 \end{array} \right. \end{aligned}$$

9 $$\begin{aligned}&B_{q}=[(3-q)\sigma _{q}^{2}]^{-1} \end{aligned}$$

In Eq. (7), the $q \rightarrow 1$ limit recovers the Gaussian distribution $P_{1}(y;\mu _{1},\sigma _{1})=\frac{1}{\sigma _{1}\sqrt{2\pi }} \exp [-\frac{1}{2}(\frac{y-\mu _{1}}{\sigma _{1}})^{2}]$ which is the basis of BG statistical mechanics. For the strongly chaotic regime where the system is ergodic and mixing with a largely positive LLE value, the Gaussian distributions are the appropriate form for the limiting probability distributions^1^. In the probability distribution analysis of the generalized standard map, for each (*W*, *K*) scenario we use $T=2^{22}$ iteration steps for $M=2\times 10^{7}$ initial conditions that are randomly chosen from the entire phase space. The distributions thereby obtained are given in the third column of Figs. 2, 3, 4, and 5 together with the corresponding phase portraits and the LLE color maps. Below, we classify the limiting probability behaviors into groups depending on the observations made on the probability distributions and we discuss each group individually.

## Results

For the $W=120$ system with $K=0.1$, and the $W=50$ systems with the rest of the *K* parameter set, due to the large amount of nonintegrability of the system, the entire phase space is fully occupied by a single chaotic sea. As the chaotic trajectories can wander through the phase space with largely positive LLE, the whole system is ergodic and mixing. For this phase space behavior, the probability distribution *P*(*y*) exhibits a clear Gaussian form, i.e., $q=1$ as expected. The phase spaces of the ($W=1$, $K=0.1$) and ($W=1$, $K=0.2$) systems, on the other hand, corresponding to the original standard map, arecdominated by stability islands making the whole system nonergodic. For initial conditions placed in these islands, the calculated LLE is nearly zero. Due to the ergodicity breakdown, the probability distribution *P*(*y*) converges to a *q*-Gaussian with $q\simeq 1.935$ as shown in Figs. 2 and 3 with parameters given in Tables 1 and 2. For the other $W=1$ systems, where the phase space contains both strongly and weakly chaotic regimes, all of the limiting probability distributions describing the whole system are obtained as a linear combination of a *q*-Gaussian with $q\simeq 1.935$ and a Gaussian function as expected from the observations made in the previous studies on the original standard map^5^. Phase space occupation ratios of these systems are determined from the Lyapunov color maps given in Figs. 4 and 5, and given in Tables 3 and 4. It should be noted that for ($W=1$, $K=0.4$) and ($W=1$, $K=0.6$) systems, the phase space occupation ratios of the stability islands were determined by detecting the constant number of initial conditions having LLE values in the $\lambda \le 4.4\times 10^{-3}$ and $\lambda \le 2.85\times 10^{-2}$ ranges, respectively.

For all map parameter values of the $W=2$ system and ($W=3$, $K=0.2$) system, a common probability distribution behavior is observed. From the Lyapunov color map of the ($W=2$, $K=0.2$) system in Fig. 3, two different strongly chaotic regions, coexisting with the stability islands, can be clearly seen with different LLE values in the phase space. By exhibiting different LLE values it can be said that the region with the larger LLE value is more chaotic than the other. To discriminate the phase space regions we utilize the Lyapunov color map by searching the range of LLE values where the phase space occupation ratios of the various initial conditions stay constant. In the analyses, we obtain $\lambda < 8\times 10^{-3}$ for the initial conditions of the stability islands, and $7.2\times 10^{-2}>\lambda > 8\times 10^{-3}$ and $7.2\times 10^{-2}<\lambda$ for the two chaotic regions. It is important to note here that the chaotic chains which can be seen in the color map belong to the strongly chaotic behavior whose chaoticity is weaker than the other chaotic sea, by lying in the same range of LLE. In our analyses, we observe that the probability distribution of the whole system is obtained as a linear combination of two Gaussians with different widths and a *q*-Gaussian, compatibly with previous observations on the portions of the phase space. This three-component probability distribution can be modeled as

10 $$\begin{aligned} P(y)=\alpha _{q_{1}}P_{q_{1}}(y;\mu _{q_{1}},\sigma _{q_{1}})+ \alpha _{q_{2}}P_{q_{2}}(y;\mu _{q_{2}}, \sigma _{q_{2}})+\alpha _{q_{3}}P_{q_{3}}(y;\mu _{q_{3}},\sigma _{q_{3}}) \end{aligned}$$

where $q_{1}\simeq 1.935$ and $q_{2}=q_{3}=1$. In Eq. (10), the contribution ratios $\alpha _{q_{1}}$, $\alpha _{q_{2}}$, and $\alpha _{q_{3}}$ are the same as the phase space occupation ratios of the initial conditions located in the stability islands, strong and less strong chaotic regions detected from the Lyapunov color map, respectively. It is clear that, by definition, $\alpha _{q_{1}}+\alpha _{q_{2}}+\alpha _{q_{3}}=1$. Strictly speaking, the contribution of the *q*-Gaussian distribution with $q\simeq 1.935$ to Eq. (10) originated from the initial conditions of the stability islands, and the initial conditions of the two different strongly chaotic regimes make contributions with two Gaussian distributions of different widths.

**Figure 6:**
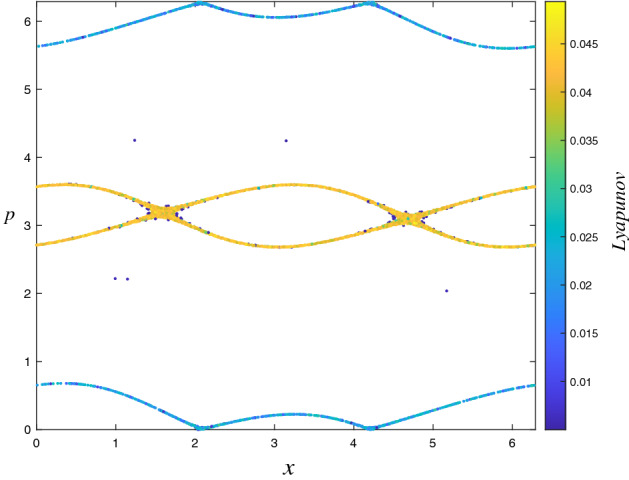
Discrimination of chaotic regions with different chaoticities that contribute two Gaussians to the probability distribution in the ($W=2$, $K=0.1$) system.

**Figure 7:**
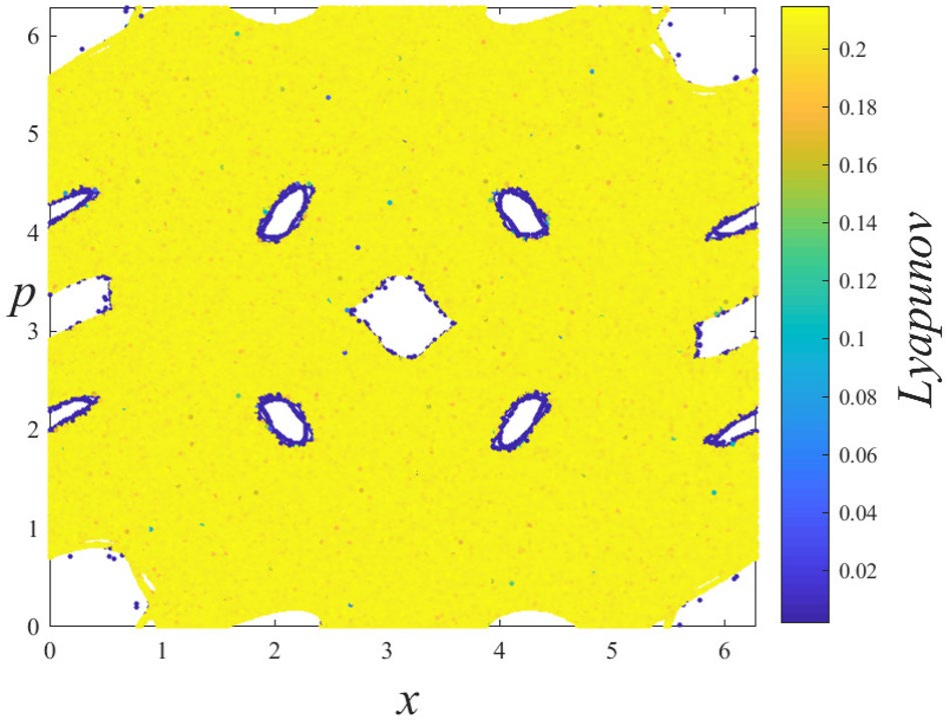
Discrimination of chaotic regions with different chaoticities that contribute two Gaussians to the probability distribution in the ($W=3$, $K=0.2$) system.

**Figure 8:**
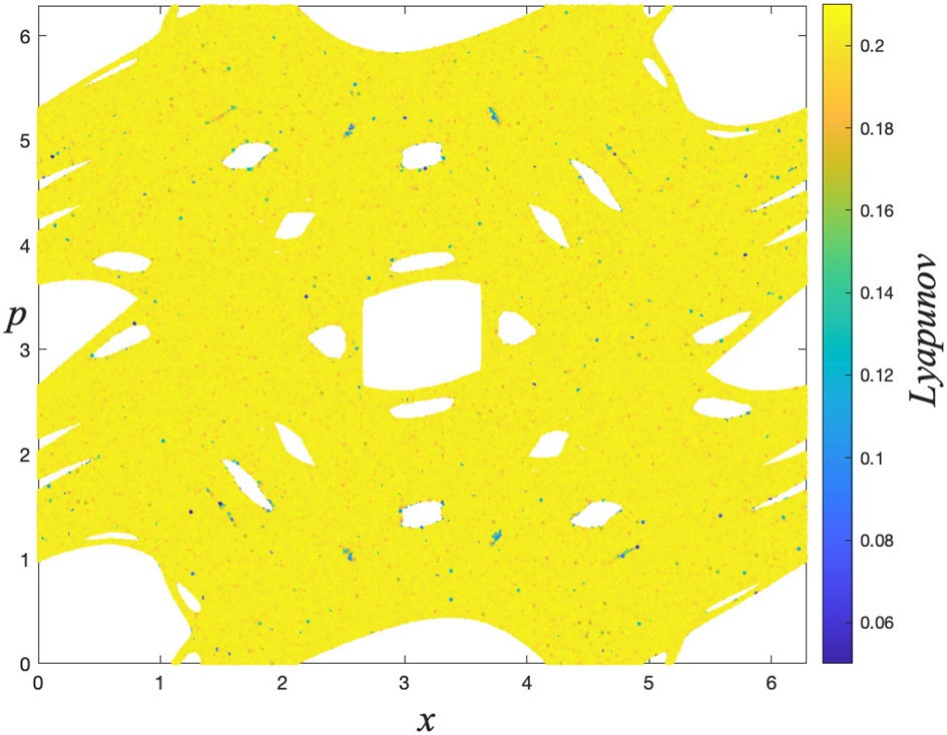
Discrimination of chaotic regions with different chaoticities that contribute two Gaussians to the probability distribution in the ($W=2$, $K=0.4$) system.

**Figure 9:**
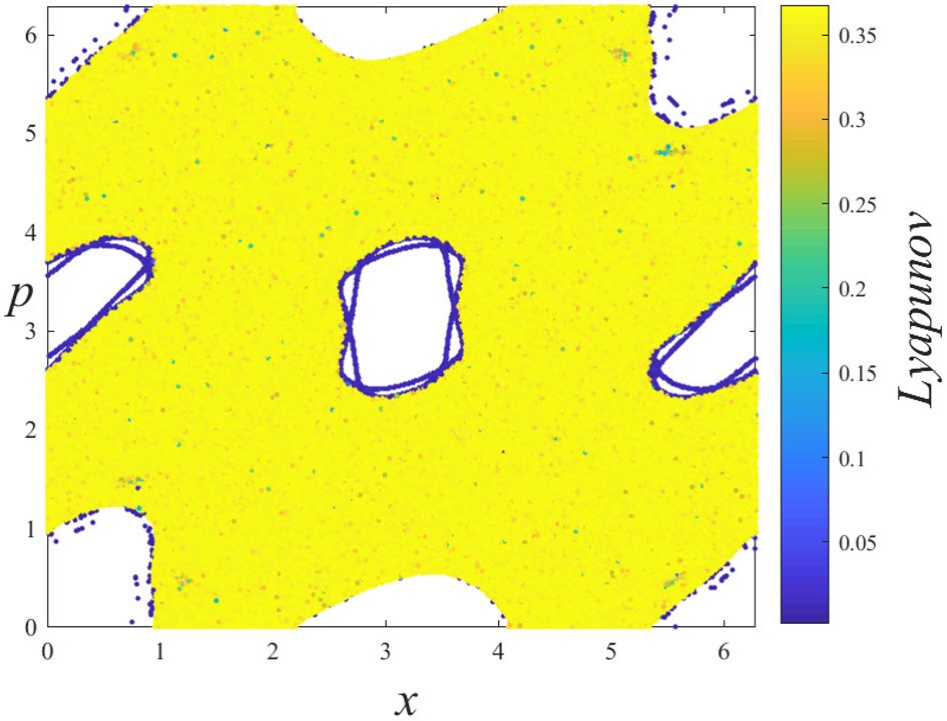
Discrimination of chaotic regions with different chaoticities that contribute two Gaussians to the probability distribution in the ($W=2$, $K=0.6$) system.

For the ($W=2$, $K=0.1$), ($W=2$, $K=0.4$), ($W=2$, $K=0.6$) and ($W=3$, $K=0.2$) cases, the phase spaces consist of both a chaotic sea and stability islands. As the system is ergodic and mixing for the strongly chaotic sea and nonergodic for the stability islands (weakly chaotic), one can predict that the probability distribution should be obtained as a linear combination of a Gaussian and a *q*-Gaussian in line with the results of recent papers^5,6^. From the obtained probability distributions, we see that this is not the case for our generalized system. Analysis of the limiting distribution of Eq. (5) by choosing a large number of initial conditions randomly from the entire phase space shows that the probability distribution can be modeled as Eq. (10), where $q_{1}\simeq 1.935$ and $q_{2}=q_{3}=1$, as in the previous case. To explain why the second unexpected Gaussian contribution appears in the probability distribution, the Lyapunov color map must be analyzed in more detail. After determining the range of LLE values for the stability islands and extracting their initial conditions from the color map, we readjust the color scale of the map to distinguish the remaining behaviors in the phase space. In addition to this visual discrimination of the phase space regions, their occupation ratios can be determined. In the ($W=2$, $K=0.1$) system, initial conditions of the stability islands having $\lambda <5.0\times 10^{-3}$ LLE values are extracted from the phase space, as shown in Fig. 6, and phase space occupation ratios of the two chaotic seas with $3.0\times 10^{-2}\ge \lambda \ge 5.0\times 10^{-3}$ and $\lambda >3.0\times 10^{-2}$ LLE are determined as $\alpha _{q_{2}}$ and $\alpha _{q_{3}}$, respectively. In Fig. 7, two strongly chaotic regimes can be clearly seen after extracting the stability islands (having LLE values in the range of $\lambda <10^{-3}$) from the phase space of the ($W=3$, $K=0.2$) system. In this figure, there are “chaotic necklaces”located inside the archipelagos bounded by the surviving KAM tori which prevent the necklaces from connecting to the strongly chaotic sea. Even though there are a few initial conditions located inside these necklaces, and the calculated LLE values are much smaller compared to the LLE values of the chaotic sea, these chaotic necklaces are statistically significant as can be understood from the probability distribution obtained. For this scenario, the phase space occupation ratios of the stability islands $\alpha _{q_{1}}$, strongly chaotic sea $\alpha _{q_{2}}$ and chaotic necklaces $\alpha _{q_{3}}$ are determined from the Lyapunov color map by detecting the constant number of initial condition ratios that present LLE values lying in the ranges $\lambda <10^{-3}$, $6.5\times 10^{-3}\ge \lambda \ge 10^{-3}$ and $\lambda >6.5\times 10^{-3}$, respectively. For the ($W=2$, $K=0.4$) system, a similar chaotic necklace structure can be observed in the LLE color map given in Fig. 8, where the stability islands with $\lambda < 2.0\times 10^{-3}$ LLE magnitudes are extracted, but with a smaller occupation ratio compared to the ($W=3$, $K=0.2$) system. In this system, chaotic necklaces have LLE values lying in the range of $1.1\times 10^{-1}\ge \lambda \ge 2.0\times 10^{-3}$, and the strongly chaotic sea trajectories exhibit the divergence of the nearby trajectories with LLE values $\lambda > 1.1\times 10^{-1}$. Again, $\alpha _{q_{2}}$ and $\alpha _{q_{3}}$ are the phase space occupation ratios of the strongly chaotic sea and chaotic necklaces, respectively. A similar chaotic necklace structure embedded inside archipelagos can be observed in Fig. 9, shown for the ($W=2$, $K=0.6$) scenario. The Gaussian with larger width arises from the initial conditions located inside the chaotic sea and the $\alpha _{q_{2}}$ contribution ratio of this distribution to Eq. (10) is equal to the phase space occupation ratio of the chaotic sea determined via $\lambda >1.5\times 10^{-2}$ LLE value range, as expected. LLE values of the initial conditions belonging to the chaotic necklaces that lie between $1.5\times 10^{-2}\ge \lambda \ge 1.5\times 10^{-3}$, and the phase space occupation ratio of these initial conditions is equal to the contribution ratio $\alpha _{q_{3}}$ of one of the two Gaussians in Eq. (10) for the limiting probability distribution. Observation of the three-component probability distribution was also made on the other generalized version of the standard map, namely the *z*-generalized standard map^24^, where the sine function is modified with an integer *z* generalization term. For both generalizations, phase spaces contain stability islands and two chaotic seas with different chaoticities and Eq. (10) successfully models the limiting probability distribution of the entire phase space. As the three-component probability distribution and the phase spaces giving rise to this distribution are newly observed in the literature, to the best of our knowledge, a more interesting observation is made on the ($W=3$, $K=0.1$) system. The phase space is occupied by the stability islands and three chaotic seas with different chaoticities that can be clearly seen in the set of graphs of this system given in Fig. 2. Phase space occupation ratios of these regions are determined from the Lyapunov color map by detecting the ranges of LLE values where the number of initial conditions remains constant. LLE ranges of different chaotic regions, with decreasing chaoticity, are detected as $\lambda \ge 8.0\times 10^{-2}$, $8.0\times 10^{-2} >\lambda \ge 5.0\times 10^{-2}$, $5.0\times 10^{-2}>\lambda \ge 3.0\times 10^{-3}$ and initial conditions that are placed in stability islands have LLE values in the range of $\lambda < 3.0\times 10^{-3}$. In line with the different regimes observed in the phase of the system, the probability distribution that characterizes the whole space is modeled as a linear combination of a *q*-Gaussian and three Gaussian functions with different widths and given as

11 $$\begin{aligned} P(y)=\alpha _{q_{1}}P_{q_{1}}(y;\mu _{q_{1}},\sigma _{q_{1}})+ \alpha _{q_{2}}P_{q_{2}}(y;\mu _{q_{2}}, \sigma _{q_{2}})+\alpha _{q_{3}}P_{q_{3}}(y;\mu _{q_{3}},\sigma _{q_{3}}) +\alpha _{q_{4}}P_{q_{4}}(y;\mu _{q_{4}},\sigma _{q_{4}}) \end{aligned}$$

where $q_{1}\simeq 1.935$ and $q_{2}=q_{3}=q_{4}=1$. The $\alpha _{q}$ contribution ratios of these functions are equal to the phase space occupation ratios determined for the regimes given above and increasing subscripts of *q* indices are assigned to the chaotic seas with decreasing occupancy and chaoticity.

The obtained parameter values of the probability distributions of the aforementioned group are provided in Tables 1, 2, 3, and 4. Here, the $B_{q}$ widths are determined numerically using the probability distributions of the each component of Eqs. (10) and (11). For the systems that exhibit stability islands (weakly chaotic) and strongly chaotic behavior in the phase space, we see that a *q*-Gaussian with $q\simeq 1.935$ is obtained for the initial conditions selected from the stability islands, and even for the large number of iteration steps, this limit behavior is as robust as the Gaussian distribution that originated from the chaotic trajectories.

**Table 1:** The obtained parameter values of the probability distributions for $K=0.1$ systems given in Fig. 2.

	$A_{q=1.935}=0.3364\dots$, $A_{q=1}=0.5642\dots$			
	$W=1$	$W=2$	$W=3$	$W=120$
$q_{1}$	1.935	1.935	1.935	1.935
$q_{2}$	1	1	1	1
$q_{3}$	1	1	1	1
$q_{4}$	1	1	1	1
$\alpha _{q_{1}}$	1	0.992	0.36	0
$\alpha _{q_{2}}$	0	0.006	0.35	1
$\alpha _{q_{3}}$	0	0.002	0.26	0
$\alpha _{q_{4}}$	0	0	0.03	0
$B_{q_{1}}$	$5.9 \times 10^{4}$	$7.9 \times 10^{4}$	$2.5 \times 10^{4}$	0
$B_{q_{2}}$	0	11	8.2	$1.5 \times 10^{-1}$
$B_{q_{3}}$	0	6.6	4.3	0
$B_{q_{4}}$	0	0	$4.9 \times 10^{-1}$	0

**Table 2:** The obtained parameter values of the probability distributions for $K=0.2$ systems given in Fig. 3.

	$A_{q=1.935}=0.3364\dots$, $A_{q=1}=0.5642\dots$			
	$W=1$	$W=2$	$W=3$	$W=50$
$q_{1}$	1.935	1.935	1.935	1.935
$q_{2}$	1	1	1	1
$q_{3}$	1	1	1	1
$\alpha _{q_{1}}$	1	0.8	0.119	0
$\alpha _{q_{2}}$	0	0.13	0.878	1
$\alpha _{q_{3}}$	0	0.07	0.003	0
$B_{q_{1}}$	$5.9 \times 10^{4}$	$7 \times 10^{5}$	$6 \times 10^{5}$	0
$B_{q_{2}}$	0	4.8	0.75	0.152
$B_{q_{3}}$	0	18	$1 \times 10^{-4}$	0

**Table 3:** The obtained parameter values of the probability distributions for $K=0.4$ systems given in Fig. 4.

	$A_{q=1.935}=0.3364\dots$, $A_{q=1.45}=0.4623\dots$, $A_{q=1}=0.5642\dots$			
	$W=1$	$W=2$	$W=3$	$W=50$
$q_{1}$	1.935	1.935	1.935	1.935
$q_{2}$	1	1	1	1
$q_{3}$	1	1	1.45	1
$\alpha _{q_{1}}$	0.9956	0.232	0.037	0
$\alpha _{q_{2}}$	0.0044	0.767	0.883	1
$\alpha _{q_{3}}$	0	0.001	0.08	0
$B_{q_{1}}$	$6.9 \times 10^{4}$	$9.0 \times 10^{3}$	$1.9 \times 10^{5}$	0
$B_{q_{2}}$	6.2	$9.0\times 10^{-1}$	$2.7\times 10^{-1}$	$1.5\times 10^{-1}$
$B_{q_{3}}$	0	3	$6.9\times 10^{-1}$	0

**Table 4:** The obtained parameter values of the probability distributions for $K=0.6$ systems given in Fig. 5.

	$A_{q=1.935}=0.3364\dots$, $A_{q=1.5}=0.4501\dots$, $A_{q=1}=0.5642\dots$			
	$W=1$	$W=2$	$W=3$	$W=50$
$q_{1}$	1.935	1.935	1.935	1.935
$q_{2}$	1	1	1	1
$q_{3}$	1	1	1.5	1
$\alpha _{q_{1}}$	0.963	0.19	0.033	0
$\alpha _{q_{2}}$	0.037	0.806	0.706	1
$\alpha _{q_{3}}$	0	0.004	0.261	0
$B_{q_{1}}$	$6.9 \times 10^{4}$	$8.0 \times 10^{5}$	$1.0 \times 10^{5}$	0
$B_{q_{2}}$	4.5	$4.8\times 10^{-1}$	$1.9\times 10^{-1}$	$1.5\times 10^{-1}$
$B_{q_{3}}$	0	$9.0\times 10^{-5}$	$3.7\times 10^{-2}$	0

As the probability distributions of the previous systems are remarkable from both statistical mechanical and chaos theory viewpoints, we encounter a more interesting situation, which was also observed in analyses of the *z*-generalized standard map^24^, for the ($W=3$, $K=0.4$) and ($W=3$, $K=0.6$) cases in Figs. 4 and 5, respectively. Even though the phase space is occupied by a strongly chaotic sea and several stability islands, the probability distribution of the whole system is modeled by Eq. (10) again, but with two different *q*-Gaussians and a Gaussian this time, namely $q_{1}=1.935$, $q_{2}=1.45$ and $q_{3}=1$ for ($W=3$, $K=0.4$), and $q_{1}=1.935$, $q_{2}=1.5$ and $q_{3}=1$ for ($W=3$, $K=0.6$). Analogously with the previous scenarios, the *q*-Gaussian with $q\simeq 1.935$ is obtained for the initial conditions chosen from the stability islands, and the $\alpha _{q_{1}}$ phase space occupation ratio is determined via the Lyapunov color map with the LLE value range $\lambda \le 10^{-3}$. Despite the analogy for the limiting behavior of the stability islands, we cannot explain the unexpected second *q*-Gaussians observed in these two systems in the same way that we explain the second Gaussian in the systems of the first group using Lyapunov color maps. Since the second Gaussians in these systems arise from the chaotic necklaces that we can distinguish as discussed above, in the present case we need to find out how this second *q*-Gaussian distribution arises. In the latter part of this paper, analyses on the ($W=3$, $K=0.6$) are given, but it should be noted that the same analyses and observations are also made for the ($W=3$, $K=0.4$) system, and they are all consistent with that of the *z*-generalized standard map^24^.

After detecting the phase space region that is responsible for the *q*-Gaussian with the $q=1.935$ contribution to the Eq. (10) and extracting this region from the phase space, only the chaotic sea presenting the largely positive LLE is left in the phase space. Thus, the Gaussian and *q*-Gaussian distributions originated from the initial conditions located in the chaotic sea. We can conclude from the observations of the previous cases and recent works^1,3–7^ that the Gaussian distribution arises from the initial conditions whose trajectories ergodically wander through the allowed energy region. As the Lyapunov color map does not provide any clue for the occurrence of the second *q*-Gaussian, we must analyze the behavior of the system in detail to clarify this observation.

**Figure 10:**
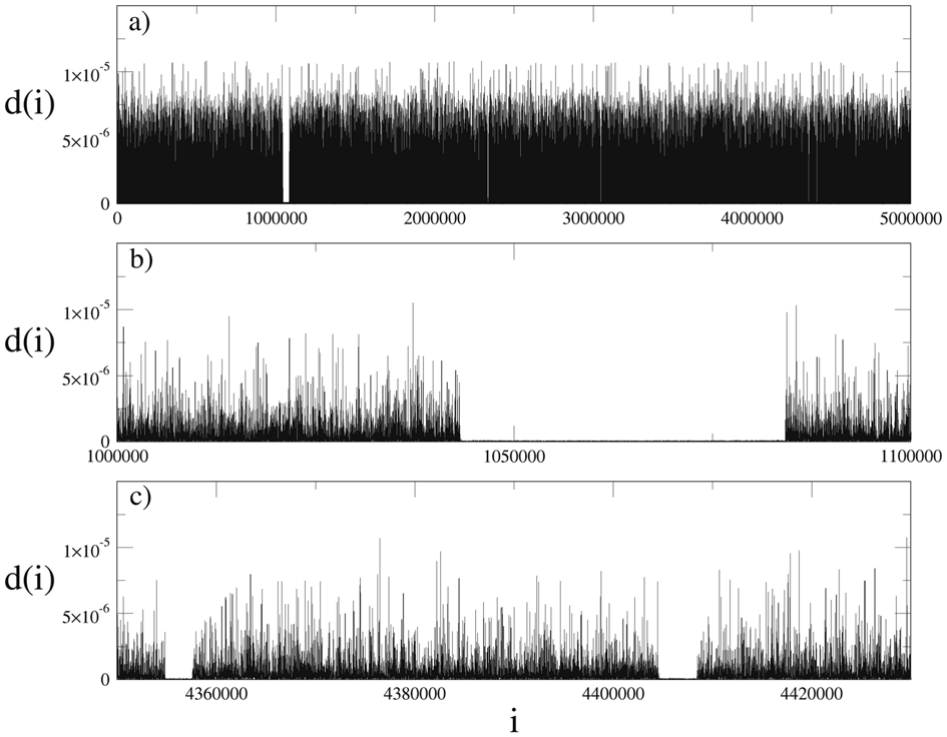
(**a**) Euclidean distance in the phase space between initially neighboring trajectories selected from the chaotic sea of the ($W=3$, $K=0.6$) system as a function of iteration step. The initial condition used here is $x=3.86735$ and $p=6.22683$. (**b**) and (**c**) Observed gaps indicate that the trajectory enters into the sticky region.

**Figure 11:**
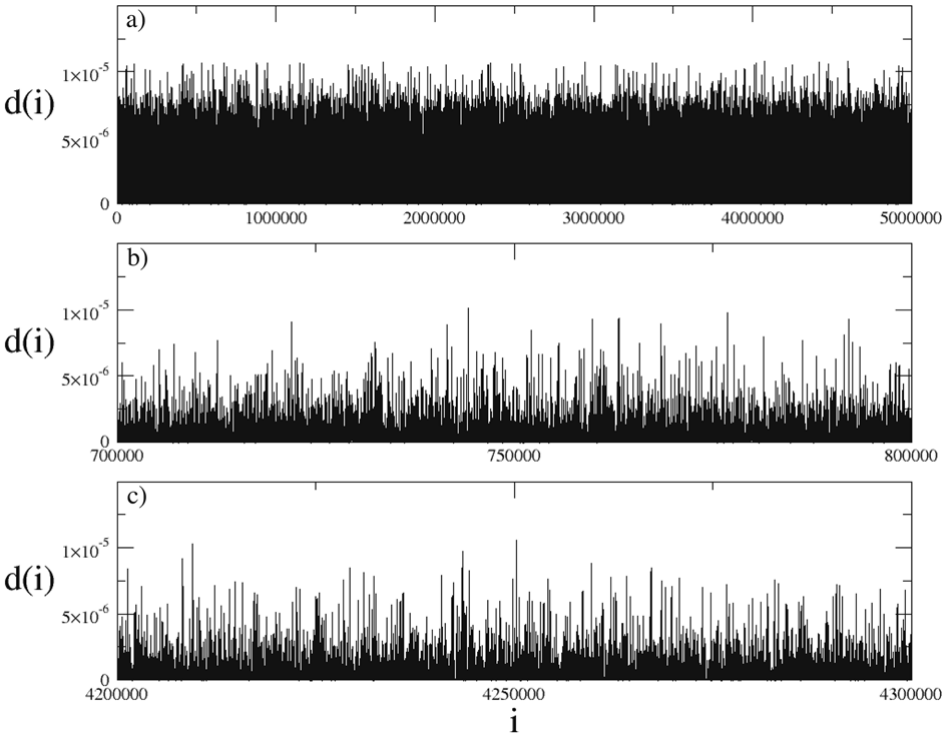
(**a**–**c**) Euclidean distance in the phase space between initially neighboring trajectories selected from the chaotic sea of the ($W=3$, $K=0.6$) system as a function of iteration step. The initial condition used here is $x=3$ and $p=3$.

In recent years it has been shown for several systems that breakdown of ergodicity and a special type of correlation are needed together for the appearance of the *q*-Gaussians. Correlations among the iterates inside the chaotic bands of the band-splitting structure that approaches the chaos threshold of the dissipative logistic map via the Huberman-Rudnick scaling law become stronger and the obtained probability distribution seems to converge to a *q*-Gaussian as this critical point is approached^4,45^. At this marginal point, the system is not ergodic or mixing. The iterates inside the nonergodic stability islands of the area-preserving maps display similar correlation behavior with those of the iterates inside the chaotic bands of the logistic map and requirements for the appearance of the *q*-Gaussians are fulfilled. It is also important to note here that in the strongly chaotic regime, the iterates of both systems wander through the phase space ergodically and exhibit uncorrelated behavior. Thus, Gaussians are the appropriate distributions for the limiting behaviors of strongly chaotic systems. If we take into account all of these factors, the unexpected *q*-Gaussian with $q=1.5$ should be understood as arising from the chaotic trajectories which behave differently compared to the regular chaotic trajectories. When we look at the phase space of the present system in more detail, we see that strongly sticky chaotic regions occur around the archipelagos. As the original standard map and other investigated cases do not exhibit such strong sticky behavior in the phase space, resonances occurring in this unique system might be the cause of the complicated tangle structures which are not observed in previous cases. The homoclinic-heteroclinic tangles are created by complex organizations of in-sets and out-sets of the hyperbolic points^18^ and we see that for the present case these tangles stick around the stability islands arising around the elliptic points located between the hyperbolic points. As KAM tori dissolve, the elliptic-hyperbolic point series exhibits specific periodicity which is related to the winding number of the dissolved torus. This structure surrounds the remaining stability islands and creates archipelagos in the phase space. For the ($W=3$, $K=0.6$) system, sticky regions occur around the archipelago which is divided into four pieces due to the modulo taken for the variables of the map. This archipelago actually develops around the identical ($x=0$, $p=0$), ($x=0$, $p=2\pi$), ($x=2\pi$, $p=0$), ($x=2\pi$, $p=2\pi$) period-1 elliptic point. Even though these sticky regions are connected to the strongly chaotic sea, from our observations we see that a trajectory starting from an initial condition located inside one of these sticky regions shows a tendency to cover the entire sticky region before escaping into the chaotic sea. As the sticky region trajectories escape into the chaotic sea after unpredictable iteration steps, the chaotic trajectories originating from the chaotic sea should eventually infiltrate the sticky regions, considering the fact that the chaotic sea is actually an equal energy region of the Hamiltonian of the standard map. In order to visualize this chaotic trajectory behavior, we utilize the Euclidean distance between two initially neighbor trajectories. In Figs. 10 and 11, we plot the Euclidean distance as a function of iteration step for initial conditions ($x=3.86735$, $p=6.22683$) and ($x=3$, $p=3$) to make comparison, respectively. For each case, the initial distance between the neighboring points is set as $\Delta _{x}^{(0)}=\Delta _{p}^{(0)}=10^{-8}$ and the system is iterated $T=5\times 10^{6}$ times, which is even larger than the number of iteration steps used in our probability distribution analyses, starting from these initial conditions. As it can be seen from Fig. 10, while the trajectory is evolving in the chaotic sea by presenting large *d* values, it enters into the sticky regions several times after unpredictable iteration steps. In this figure, the distance values decrease abruptly by creating gaps, indicating that the trajectory is wandering in the confined sticky region and covering it. Even though the distance values are small, initially neighboring trajectories diverge exponentially in sticky regions as expected from the chaotic trajectories. On the other hand, distance values given in Fig. 11 do not exhibit decrement gaps and trajectory wanders through the chaotic sea without entering into sticky regions during iteration steps used in the calculation. When we look at the Lyapunov color map given in Fig. 5 for the present system, we see that initial conditions located inside sticky regions and the chaotic sea exhibit similar LLE values. Even though the Euclidean distances are very small while the trajectory is in the sticky regions, large distance values originating from the trajectory movement in the chaotic sea repress the contribution of the smaller distance values in the summation of logarithmic functions in Eq. (4) and we obtain largely positive LLE values. This observation does not indicate that the calculation is incorrect or that some dynamics are ignored. For Lyapunov exponent calculation in the theory of nonlinear dynamics, making long-term observations for a trajectory instead of short-term observations, and obtaining the exponential value which characterizes the long-term behavior of the trajectory is an accurate calculation^18^. By comparing these figures, we conclude that whereas some trajectories may enter into the sticky regions and stay in there for many iteration steps, other trajectories may not visit these regions at all during the observation time. These two different trajectory behaviors might be a good explanation for the two distributions obtained in the limiting behavior analyses, i.e., Gaussian and *q*-Gaussian with $q=1.5$. Chaotic trajectories which wander through the chaotic sea without entering into the sticky regions exhibit exponential divergence of initially nearby trajectories with large LLE values, and they spread into the chaotic sea with apparently random behavior indicating the mixing property. The Gaussian distribution obtained from the initial conditions located inside the chaotic sea of the present system is thought to originate from these trajectories. On the other hand, when the trajectory enters into the sticky region, it follows a similar phase space path to that of the archipelago while it is covering the sticky region. As the sticky region surrounds the archipelago and stability islands, it exhibits similar spreading behavior in the phase space to that of the archipelago due to the tangle structure mentioned above. While a chaotic trajectory is covering the sticky region, it does not spread into the most of the allowed energy region for many iteration steps and it also does not exhibit apparently random behavior like the regular chaotic trajectories present. By considering this spreading behavior, we can conclude that chaotic trajectories which enter into the sticky regions will not exhibit mixing property during the iteration process. One last important issue to note here is that, for an infinitely long time (number of iterations), each chaotic trajectory will eventually cover the entire allowed region. However, if we consider the computation limits in simulations of real world systems, this limit cannot be reached and the number of iteration steps used in this paper is reasonable for characterizing the limiting behavior as shown in previous papers^5–7^. As a consequence of the lack of the mixing property, these trajectories can be related to the observed unexpected *q*-Gaussian for the present case. At this point, it is worth mentioning that stickiness and detection of sticky regions have already been studied in the literature intensively^46–48^.

**Figure 12:**
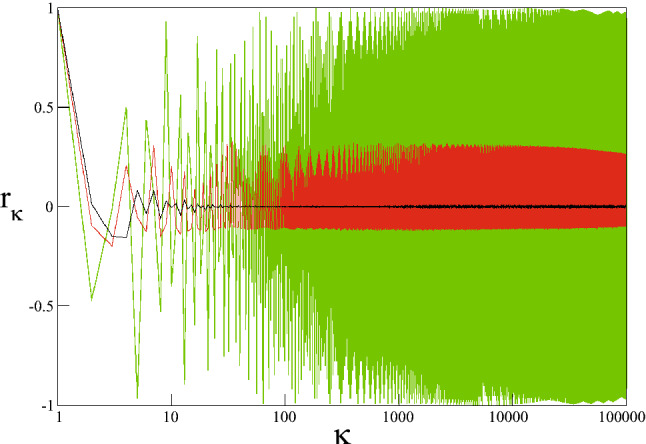
Auto-correlation functions of data points obtained for initial conditions selected from the stability island (green) and the chaotic sea (red and black) of the ($W=3$, $K=0.6$) system.

Focusing on the chaotic trajectory spreading behavior in the sticky regions and considering the second requirement for the appearance of the *q*-Gaussians, we can investigate correlations among the iterates of a trajectory. At this stage, we investigate the correlations among iterates by regarding trajectories as time series. We use the auto-correlation function $r_{\kappa }$ in order to analyze whether the data of a time series is correlated. This function is defined as

12 $$\begin{aligned} r_{\kappa }=\sum _{i=1}^{T-\kappa }\frac{(y_{i}-\langle y\rangle )(y_{i+\kappa }-\langle y\rangle )}{\sum _{i=1}^{T}(y_{i}-\langle y\rangle )^{2}} \end{aligned}$$

where $\langle y\rangle =T^{-1}\sum _{i=1}^{T}y_{i}$ is the mean, *T* is the total number of data points of the time series and $\kappa$ is the time lag^44^. The data of the time series is said to be correlated if $r_{\kappa }\ne 0$ and not correlated if $r_{\kappa }=0$ for $\kappa \ge 1$. For the present scenario, time series are created starting from numerous initial conditions chosen from the chaotic sea by using the same $T=2^{22}$ used in the computation of probability distributions. As large numbers of initial conditions are chosen randomly from the chaotic sea, we also choose a few initial conditions located in the stability islands in order to be able to make comparisons and provide an example of the suggested correlated behavior among iterates located in the stability islands in Ref.^5^. By considering calculation times and the time required to analyze each time series, we decided to use $\kappa =10^{6}$ as an upper limit for the time lag. As a result of the analyses, it was observed that there are three cases that can be observed for auto-correlation functions of the times series of the initial conditions selected from the phase space of the ($W=3$, $K=0.6$) system and results are given in Fig. 12. From this figure it can be seen that the green curve, obtained for the time series starting from ($x=5.800745\dots$, $p=5.980580\dots$), located in a stability island, oscillates around zero with very large fluctuations, indicating the correlated nature of the data. Moreover, the black curve immediately decreases to zero and oscillates in the vicinity of zero, as a consequence of uncorrelated data. This curve is computed for the time series obtained from the iteration of the ($x=6.275676\dots$, $p=6.104209\dots$) initial condition located in the chaotic sea. Green and black signals exhibit oscillating behavior similar to that of the auto-correlation functions calculated for the iterates of the logistic map and for the white noise in Ref.^4^, respectively. Whereas the behavior of the black auto-correlation function is understandable if we consider the apparently random behavior of the strongly chaotic trajectory^18^, the red auto-correlation function presents unexpected behavior for a chaotic trajectory. It oscillates around zero with large fluctuations that are significantly larger than those of the black curve and smaller than those of the green curve. This auto-correlation function behavior indicates that the data of the time series used in the computation is correlated. The initial condition ($x=5.815235\dots$, $p=5.523288\dots$), for which red-colored auto-correlation function is obtained, is located inside the sticky region. As the whole chaotic sea is an allowed energy surface created by dissolution of the stability islands arising due to the increment of the nonintegrability of the system, these sticky regions are also a part of this chaotic sea. A chaotic trajectory of the chaotic sea has to visit sticky regions as the system evolves in time, and that trajectory will enter sticky regions and escape back to the chaotic sea. As we see from the auto-correlation functions, however, some trajectories which arise in sticky regions may exhibit correlations which are weaker than those in the stability islands and stronger than those of the regular chaotic trajectories. The observed correlated nature of the chaotic trajectories is significant from both the theory of nonlinear dynamics and the statistical mechanical viewpoints. Here we suggest that these three kinds of auto-correlation functions might be directly related to the three-component probability distribution obtained for the ($W=3$, $K=0.6$) system. Together with their phase space behavior mentioned above, the initial conditions give rise to green, red and black signals with decreasing correlations, and those might be responsible for the $q_{1}=1.935$, $q_{2}=1.5$ and $q_{3}=1$ distributions in Eq. (10), respectively. In other words, the stronger correlation property is related to the larger *q* value for the present system. As the contribution of the $q_{1}=1.935$ distribution is also verified via the phase space occupation ratio detected from the Lyapunov color map, we are able to determine the contribution ratios of the other two distributions numerically only by using the obtained probability distribution as a consequence of the unpredictable phase space behavior of the chaotic trajectories of the present system.

## Discussion

The results obtained in this paper have great potential importance in diverse fields of physics and also in Hamiltonian chaos theory investigations by considering the examples given in the “Introduction” section for the original standard map. The present modification of the standard map enables us to create unique area-preserving maps derived from the Hamiltonian with multiple-well potential. For each scenario analyzed in this paper, a *q*-Gaussian distribution with $q\simeq 1.935$ is obtained for initial conditions chosen from the stability islands. Together with the results of the recent papers^6,7^, observations made for the generalized standard map can be considered as an indication of the robustness of this specific *q*-Gaussian for the stability islands of the area-preserving maps. Even for the large number of iteration steps used in our simulations, this limiting behavior is maintained together with those of the other distributions, as can be understood from the obtained linear combination form for the probability distributions. As these probability distributions describe the entire system, which consists of different behavior regions with different phase space occupation ratios for each case, we also observe effects of the generalization made for the standard map. In some scenarios, the second and even third chaotic region occurs together with strongly chaotic sea and stability islands. For these cases, the limiting probability distribution is obtained as a linear combination of a *q*-Gaussian with $q\simeq 1.935$ and Gaussians with different widths that are related to the number of regions in the phase space. Since this multi-component probability distribution can be understood by considering different behavior regions, we encounter a complicated limit behavior for the ($W=3$, $K=0.4$) and ($W=3$, $K=0.6$) systems which exhibit strong sticky regions. For these cases, the sticky regions that occur around archipelagos cause chaotic trajectories to behave in a different way from that expected for the regular chaotic trajectories. As chaotic trajectories starting from sticky regions may not spread into the allowed energy surface apparently randomly during iteration process used in limiting distribution analyses, these chaotic trajectories also exhibit a correlated nature. These revealed properties of the sticky region chaotic trajectories are thought to be directly related to the obtained *q*-Gaussian with $q=1.5$ in the above-mentioned system. Even if the probability distribution of the sticky region chaotic trajectories should converge to a Gaussian in the limit of infinite time, considering the unreachability of this limit in numerical calculations or in physical applications, one can conclude that this observation should be valid not only for the present case also for other area-preserving maps that exhibit such strong sticky behavior in their phase spaces.

## Data Availability

The datasets generated during and/or analysed during the current study are available from the corresponding author on reasonable request.
